# Osteosynthesis in Osteogenesis Imperfecta, telescopic versus non-telescopic nailing

**Published:** 2015

**Authors:** A Sterian, R Balanescu, A Barbilian, A Ulici

**Affiliations:** * ”Grigore Alexandrescu” Clinical Emergency Hospital for Children, Bucharest, Romania; ** ”Carol Davila” University of Medicine and Pharmacy, Bucharest, Romania; *** ”Dr. Carol Davila” Central Military University Emergency Hospital, Bucharest, Romania

**Keywords:** Osteosynthesis, Osteogenesis Imperfecta, telescopic versus non-telescopic nailing, Fassier–Duval nails

## Abstract

The paper refers to a pediatric patient suffering from Osteogenesis Imperfecta that was diagnosed soon after birth, after suffering from an intrauterine fracture of the femur in the 7th month of pregnancy. The beginning of the presentation contains some general considerations regarding the illness and the treatment done up to the point when the first telescopic rod was used. Following the evolution of the child from birth to the age of 7 years, we could trace a line of evolution under several methods of treatment, surgical or conservative, and also on different surgical treatment variants and their outcome during growth. Together with the X-rays that documented each step of the treatment, we could affirm for sure that both clinically and radiologically, the best results were obtained after the last 4 interventions, when all 4 major bones of the lower limbs were operated on.

Until the moment Fassier–Duval nails were used, the evolution of the illness and the complications that appeared after certain surgery procedures were not so good. Several procedures had to be revised because of nail or pin displacement and eventually the patient lost the walking capability. The main problem with non telescopic treatment was the lack of stability that the bone needed to have after an open surgery for deformity correction, and up to that moment, the methods used were not designed to work on the long term; even in the best circumstances, the patient had to go to the OR for nail replacement after the bone outgrew it.

## Introduction

Osteogenesis Imperfecta (OI) is a hereditary condition affecting approximately 1 in 20,000 births with eleven recognized types of illness each one with its own characteristics [**1**-**3**]. Children with OI have low bone mass together with poor structural value, so, these two problems lead to recurrent fractures, varying degrees of short stature and progressive deformities of the limb and of the whole body in general. Severity ranges from those mildly affected individuals (Type 1) who have minimal bone deformity, near normal stature, blue sclerae and variable hearing loss, to those who are lethally affected with multiple fractures both in utero and in the first month of life that lead to respiratory failure and death [**2**,**4**,**5**].

On top of all these problems, the most important and dramatic one regards the bone system, and, the way it manifests itself is by brittleness of the bones that lead to more and more fractures as the child grows, the peak being in the first years after the walking age, that is about one year and 2 months to 1 year and 6 months. In all the cases this is the point when something must be done immediately because the prognosis goes from worse to worst as time goes by when the child grows and begins to walk, thus limiting the child capacity for a normal life. The main drawback of not doing something in order to stabilize the bones and reinforce them is the development of progressive deformities of the bone that add up more stress on the child, parents and eventually on the medical staff because surgery gets riskier and more complex [**6**,**7**]. 

The article deals with the case of a patient diagnosed with Osteogenesis Imperfecta at the age of 3 months. By that time, he already suffered 3 left femur fractures, one of which was at the age of 7 months, in utero, being accidentally diagnosed during a checkup the mother had at that time. Until the age of 4 years, the patient suffered 8 fractures of the lower limbs, all of them located at the level of the femurs, evenly distributed amongst right and left. Until that age, the treatment was done solely orthopedically. No cast immobilization and surgery were undergone to correct the bone deformity that developed or any kind of osteosynthesis.

The first surgical procedure was made at the age of 4 years and 3 months when a Kirschner wire was used to stabilize the left femur after multiple corrective osteotomies and cast immobilization, procedure that had a poor outcome as the wire slipped after 3 weeks destabilizing the osteotomies and putting the soft tissue at risk. After this episode, a revision surgery was carried out to remove the wire and replace it with a Rush nail, that proved to have good results until the age of 5 when the distal end of the rod punctured the cortical bone of the femur and produced severe discomfort and eventually got replaced by a larger Rush nail. The same problems occurred for the right femur when the first operation was made, in this case 2 revisions were made to change 1 rush nail and one Küntscher nail. Eventually, the quality of the bone increased due to bisphosphonate treatment, and no new fractures occurred at the femoral level. Cast immobilization was used postoperatively for each of the surgeries following the basic protocol for lower extremities fractures, without any special rules. The limb was immobilized for 6 weeks, and full weight bearing was resumed 4 weeks after cast removal.

**Fig. 1:**
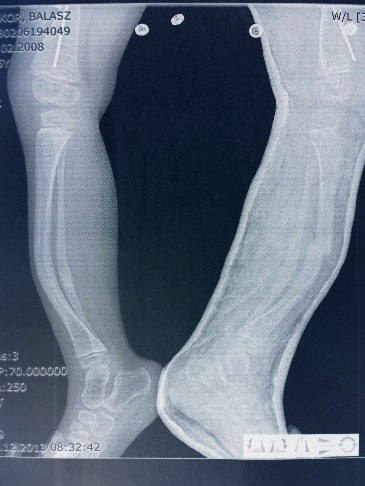
X-ray showing severe bowing of both tibias (approx. 300). Left tibia has a displaced diaphyseal fracture produced while walking

**Fig. 2:**
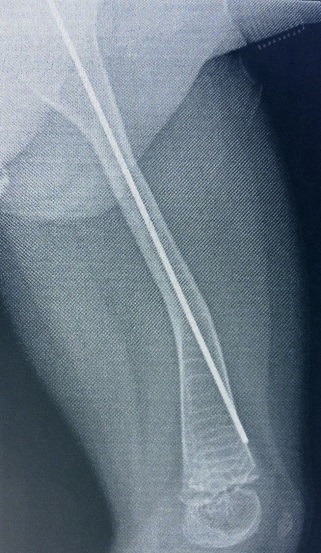
Kirschner wire migrating through distal cortical wall in a femur. Secondary bowing of the bone marginal to the distal end of the wire

**Fig. 3:**
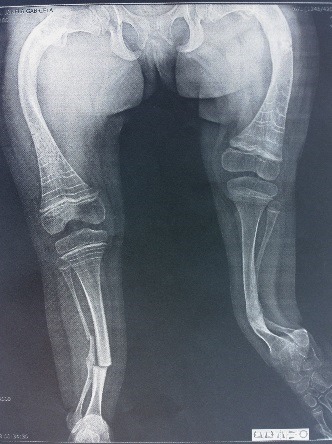
Severe bowing of the lower extremities. Both femurs have near 900 angulation after several fractures treated by cast immobilization. Right tibia fracture with displacement. Left tibia and peroneum fractures badly consolidated at 900 after a poor orthopaedic management

**Fig. 4:**
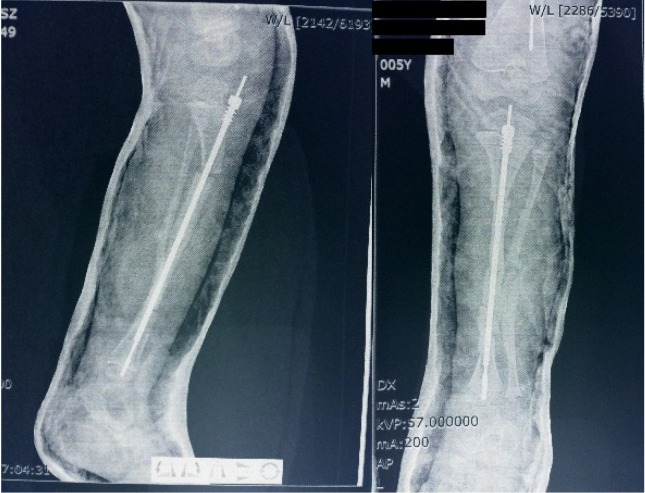
Postoperative image showing Fassier-Duval telescopic rod and corective osteotomies of the tibia

**Fig. 5:**
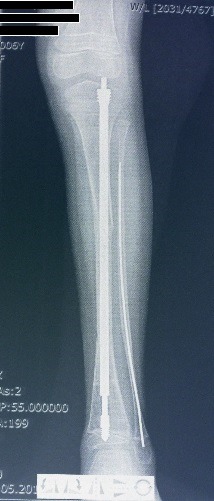
Late post surgery image of the same tibia with good bone healing, no component migration, and good bone alignment

**Fig. 6:**
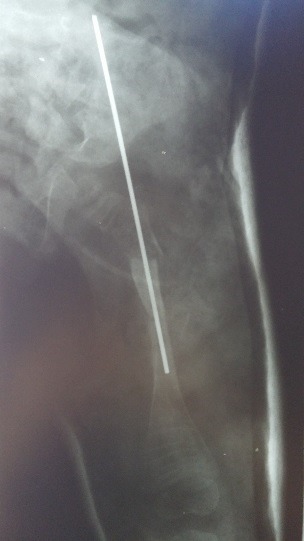
Femur corective osteotomies and Kirschner wire ostesinthesys. Note the proximal displacement of the wire and iminent loss of stabilitiy

By the age of 5, no tibia fractures occurred, but as the child began to walk, the bones bowed more and more up to 300 of anterior angulation, the point when the first fracture occurred at the level of the left tibia. At that point, the decision was made to try the Fassier-Duval telescopic nail system in order to correct the deformity and try a more stable and reliable osteosynthesis on long term.

The procedure presupposed that, for the purpose of correction, 5 osteotomies had to be made at the diaphyseal level followed by the reaming of the fragments in order to get the nail through [**8**]. The corrective procedure follows the Soffield–Millar protocol for the correction of severe deformities of long bones, and was chosen because at the moment of surgery the angulation was very big and no medullar canal was available for nail insertion through percutaneous techniques or minimally invasive osteotomies described in the original Fassier–Duval technique [**9**].

In the upcoming 2 years, the child had 3 more surgeries for the right tibia and both femurs. After the surgeries no complications occurred, weather serious or less serious. And, most importantly, the patient regained ambulation, he began walking unaided, run, jump and have a normal life. In all 4 cases, the osteotomies healed normally without any delayed union of pseudarthrosis, the nail components worked perfectly, all 4 maintaining the alignment as the bone grew in length, without any loosening appearing in the canal or any signs of growth plate damage seen at that point, the scars healing normally.

## Conclusion

To summarize, it can certainly be stated that Fassier-Duval telescopic nail is the best answer to Osteogenesis Imperfecta patients with multiple fractures and severely deformed long bones. Unfortunately, such cases are still being reported and present to the hospital with great delay, the method mentioned above showing exactly the outcome of non-telescopic nail treatment and its outcome during childhood when the natural growing mechanism represents a challenge that needs to be overcome in order to get the best results. The differences between the evolution after non-telescopic nails and telescopic nails are very clear in the overall picture, having a far better evolution post surgery, after telescopic nailing. Ambulation and social integration is the key goal for a pediatric patient and following this protocol gives the child the chance to have a life as close to normal as possible. 
